# Na/K-ATPase in the renal proximal tubule cell: classic & novel roles in sodium handling

**DOI:** 10.3389/fphys.2026.1858622

**Published:** 2026-08-07

**Authors:** Kristiana Sklioutovskaya-Lopez, Maafi R. Islam, Shreya T. Mukherji, Sidney Strause, John M. Crutchley, Shuyan Sun, Marco T. Pessoa, Sandrine V. Pierre

**Affiliations:** 1 Department of Biomedical Sciences, Joan C. Edwards School of Medicine, Marshall University, Huntington, WV, United States; 2 Aera Therapeutics, Cambridge, MA, United States

**Keywords:** ATP1A1, cardiotonic steroids, kidney, ouabain, salt-sensitive hypertension, signaling

## Abstract

The cell membrane Na/K-ATPase (NKA), the archetypal P-type ATPase, is the enzymatic complex that hydrolyzes ATP to transport Na^+^ and K^+^ against their transmembrane electrochemical gradients. While it has long been known that NKAs are essential to homeostasis in animal cells, active research continues to unveil the depth and complexity of their tissue-specific functions as ion-pumps and receptors for endogenous cardiotonic steroids (eCTS). Sodium handling in the renal proximal tubule (RPT), a major regulator of systemic salt and water balance and blood pressure, is among the cell-specific NKA functions that have been the most studied to date. A rather comprehensive and integrated view of the biological pathways involved has emerged in physiological conditions and in disease states such as salt-sensitive hypertension and chronic kidney disease. Increasingly, sex-specific differences in the distribution and activity of NKA and other transporters along the nephron are reported, with functional links to differences in sodium handling and disease susceptibility. Following a brief overview of NKAs’ molecular composition and functions, this review focuses on the molecular structure, regulatory mechanisms, and classic and novel roles of NKA in the RPT, emphasizing its importance in sodium handling, blood pressure regulation, and renal disease.

## Introduction

In the mid-1950s, Jens. C. Skou first identified the membrane Na/K-ATPase (NKA) protein as the “sodium pump”. This archetypal P-type ATPase is the enzymatic complex that hydrolyzes ATP and is specifically activated to transport Na^+^ and K^+^ against their electrochemical gradients at physiological intracellular Na^+^ and extracellular K^+^ concentrations (Skou, 1957). Key molecular features of NKAs include their heteromeric subunit composition and distinct binding sites for ions, ATP, and regulatory molecules, including specific ligands of the cardiotonic steroids family (CTS). While it has long been known that NKAs are essential to homeostasis in animal cells, active research continues to unveil the depth and complexity of NKAs’ cell-specific functions as ion-pumps and receptors for endogenous CTS (eCTS). Sodium handling in the renal proximal tubule (RPT) is among the cell-specific NKA functions that have been the most studied to date. A rather comprehensive and integrated view of the biological pathways involved has emerged in health and diseases and is the focus of this review. While we propose a brief overview of the general features of CTS/NKAs across mammalian organ systems as an *Introduction*, the more comprehensive in-depth review by Trant et al. (and references therein) in this Special Issue “*The Sodium Pump and Cardiotonic Steroids in Health and Disease*” is recommended (Trant et al., 2025).

**Figure 1:**
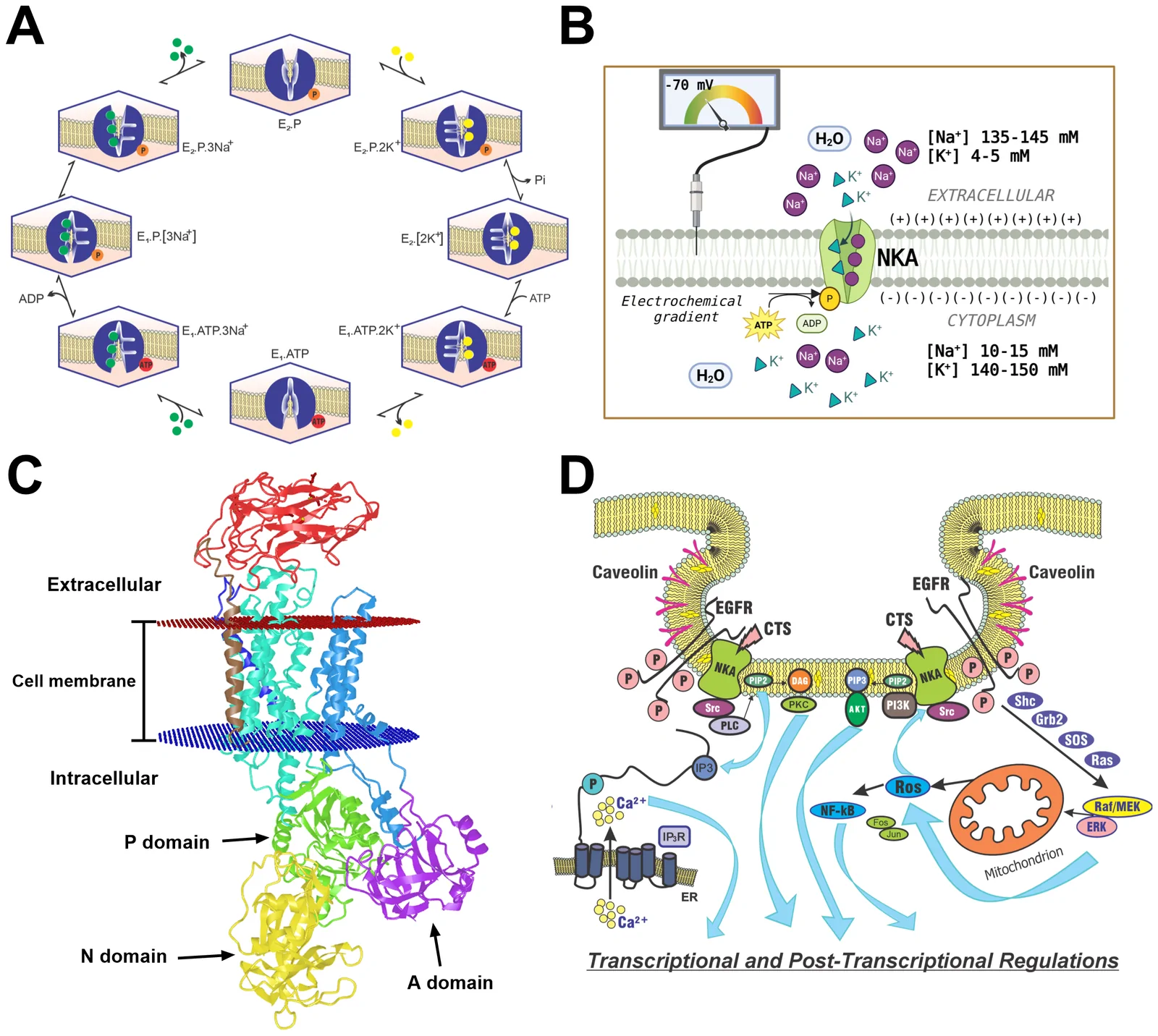
Fundamental features of Na/K-ATPase (NKA). **(A)** The Albers-Post reaction mechanism. Three sodium ions (green circles) bind to NKA in E1.ATP conformation (E1.ATP.3Na^+^). Upon hydrolysis of one ATP molecule (red circle), the enzyme is phosphorylated (orange circle) on a conserved aspartate residue, and three sodium ions are occluded (E1.P.[3Na^+^]). A conformational change to E2.P.3Na^+^ occurs. The sodium ions are released into the extracellular space, and two potassium ions (yellow circles) bind to E2.P (E2.P.2K^+^). Dephosphorylation followed by occlusion of the potassium ions occurs in [E2.(2K^+^)]. Finally, one ATP binds to the enzyme, another conformational change occurs (E1.ATP.2K^+^), and the two potassium ions are released into the cytosol (E1.ATP). **(B)** The enzymatic activity of NKA maintains the Na^+^ and K^+^ transmembrane electrochemical gradients that are fundamental for cell volume, intracellular pH, and overall homeostasis. These gradients are required for the coupled transport of ions and molecules, as well as the resting membrane potential. **(C)** NKA crystal structure (PDB ID: 2ZXE) from shark (*Squalus acanthias*) rectal gland represented in ribbon style using iCn3D (Wang et al., 2020). The α subunit transmembrane domains (cyan and blue), the actuator domain A (magenta), the phosphorylation domain P (green), and the nucleotide-binding domain N (yellow) are shown. The β subunit is represented in red, with its unique transmembrane domain partially hidden in the back of the tridimensional structure (dark blue). A small polypeptide of the FXYD family is shown in brown. **(D)** The NKA signalosome. CTS binding to NKA initiates tissue-specific signaling cascades. NKA functions as a scaffolding hub that organizes multiple signaling partners, including Src kinase, PI3K/Akt, and reactive oxygen species (ROS). These coordinated interactions give rise to highly regulated, context−dependent downstream pathways that influence cell survival, metabolism, proliferation, and differentiation. (Adapted from Cui and Xie, 2017).

NKAs were first identified as plasma membrane ion pumps that utilize the energy produced from hydrolyzing the terminal phosphate of one ATP molecule to transport 3 Na^+^ out of the cell and 2 K^+^ into the cell per enzymatic cycle. To transport cations against their concentration gradients, binding sites with locking gates occlude their movements in the opposite, energetically favorable direction following conformational transition to two main conformations: E1 (which becomes phosphorylated in the presence of ATP) and E2 (which is dephosphorylated in the presence of K^+^) as described in the Albers-Post scheme (**Figure 1A**) (Albers, 1967; Post et al., 1972; Kaplan, 2002). By establishing and maintaining the transmembrane electrochemical gradients of Na^+^ and K^+^ in every cell, NKAs set the cell resting membrane potential and drive secondary transport of other ions, nutrients, and water. Hence, NKAs’ ion-pumping function is critical for the maintenance of cell volume, pH, and overall homeostasis, as well as for the generation of action potentials in excitable cells (**Figure 1B**) (Tosteson and Hoffman, 1960; Armstrong, 2003; Kay and Blaustein, 2019).

NKAs are oligomeric protein complexes typically composed of three subunits: α, β, and an accessory regulatory protein of the FXYD family as illustrated in **Figure 1C**. The α subunit is the large, multi-transmembrane catalytic component of the enzyme which contains the binding sites for Na^+^, K^+^, ATP, and specific ligands such as CTS. NKA α subunits have 10 transmembrane helices and a large cytoplasmic domain that contains the signature aspartate residue temporarily phosphorylated during the catalytic cycle in all P-type ATPases. In mammals, four isoforms have been reported (α1-4, encoded by *ATP1A1-4*), each with unique tissue-specific expression patterns. Alpha 1 is expressed ubiquitously. Alpha 2 is predominantly found in muscle (smooth, skeletal and cardiac) and in glia (Zahler et al., 1992; Cameron et al., 1994; Lavoie et al., 1997). Alpha 3 is expressed in neurons, and α4 is found specifically in the testis (Hieber et al., 1991; McGrail et al., 1991; Shamraj and Lingrel, 1994; Blanco and Mercer, 1998). The β subunit is a single-transmembrane, heavily glycosylated protein that is crucial for the proper folding, assembly, and trafficking of NKA to the cell membrane. There are three known mammalian isoforms, β1-3, that modulate the enzyme’s kinetic properties and can function as cell-adhesion molecules. Beta 1 is widely expressed, and important for epithelial cell adhesion (Vagin et al., 2012). Beta 2 is expressed in the nervous system, where it mediates adhesion between glial cells and neurons (Gloor et al., 1990; Roldan et al., 2022). Beta 3 mRNA and protein are expressed in the lungs, testis, liver, and skeletal muscles (Malik et al., 1996; Arystarkhova and Sweadner, 1997). Functional properties of NKA αβ oligomers are also modified through association with protein members of the FXYD family. FXYD proteins share a 35-amino acid signature sequence, beginning with the amino acid sequence PFXYD (Yap et al., 2021). In mammals, seven isoforms of FXYD have been reported, with distinct tissue distributions. All seven members of the family have been shown to act as auxiliary NKA subunits and alter the enzymatic activity of the NKA complex (Lubarski et al., 2005; Geering, 2006; Delprat et al., 2007; Lubarski-Gotliv et al., 2016; Meyer et al., 2020). FXYD1 (phospholemman, PLM) is expressed predominantly in the heart, skeletal muscle, and brain (Bogaev et al., 2001). FXYD3 (Mat-8) is overexpressed in certain types of cancers. FXYD6 (phosphohippolin) and FXYD7 are mainly expressed in the nervous system (Béguin et al., 2002; Geering, 2006; Delprat et al., 2007). FXYD4 (Aggarwal et al., 2024) is localized in the renal collecting duct where it increases NKA sodium affinity and activity (Geering et al., 2003). FXYD5, which enhances NKA activity by increasing its Na^+^ affinity or V_max_, has been detected in the spleen, intestines, and in the collecting duct and connecting tubules of the kidney (Lubarski et al., 2005; Arystarkhova et al., 2007). FXYD2 (NKA γ subunit) is primarily expressed in the kidneys where the bulk of Na^+^ reabsorption occurs. FXYD2 exists as two splice variants (FXYD2a and FXYD2b). In the RPT, the FXYD2a variant is the major form, while FXYD2b predominates in the distal convoluted and connecting tubules. Both variants are present in the thick ascending limb (Pu et al., 2001; Arystarkhova et al., 2002a, 2002). Functionally, both splice variants increase NKAαβ affinity for ATP and decrease affinity for Na^+^, supporting optimal Na^+^ reabsorption (Arystarkhova et al., 2002a). Together, FXYD2 and FXYD4 establish a gradient of NKA Na^+^ affinity along the nephron, with a lower affinity proximally and higher affinity distally, to enable adaptive regulation of Na^+^ balance (Yap et al., 2021). The first report on the clinical consequences of a pathogenic variant of FXYD2a was recently published by Zawadzki et al., who described a missense substitution in the FXYD2a gene that resulted in Na^+^-dependent reduced hexose absorption by the RPT and overall volume-dependent tubular transport disorder (Zawadzki et al., 2025).

Cardiotonic steroids (CTS) such as ouabain and digoxin are specific and potent inhibitors of NKA’s enzymatic function, which is the molecular basis for their toxicity and historical use as poisons (Fraser, 1872; Katz, 1985; Shrestha et al., 1992). Within their margin of safety, CTS are used in the clinical treatment of Cardiac arrhythmias, including atrial fibrillation, atrial flutter, and paroxysmal supraventricular tachycardia (Yang et al., 2012; Whayne, 2018). Chronic administration of CTS such as digoxin or digitoxin also show efficacy in the management of heart failure with reduced ejection fraction (HFrEF), which contrasts with other positive inotropic agents (Gibbs et al., 2000; Whayne, 2018; Bavendiek et al., 2025; Geavlete et al., 2026). While CTS from plants and non-mammalian vertebrates have been described for centuries (Norn and Kruse, 2004; Wang et al., 2017; Qi et al., 2018), their presence in mammalian tissue is a relatively recent development (Flier, 1978; Gruber et al., 1980; Lichtstein and Samuelov, 1980; Hamlyn et al., 1991; Lichtstein et al., 1993; Bagrov et al., 1998; Komiyama et al., 2005; Blaustein and Hamlyn, 2024). Since then, the biological role of eCTS has been the subject of intense investigation, leading to the current understanding of the eCTS/NKA hormone/receptor system (Aizman et al., 2001; Scheiner-Bobis and Schoner, 2001; Goldstein et al., 2012; Dvela-Levitt et al., 2015; Hodes et al., 2016; Blaustein and Hamlyn, 2020). Mechanistically, we have learned that CTS binding to the extracellular domain of NKA can initiate cell-specific responses without measurable change in cellular ion homeostasis (Liu et al., 2000; Xie and Askari, 2002; Liu and Xie, 2010; Blaustein and Hamlyn, 2024). Several common features of CTS signaling through NKA, which occur without measurable change in cellular Na^+^/K^+^-ATPase activity, have been described at the molecular level. They include changes of conformation, protein-protein interaction, and cellular distribution, as well as activation of protein kinases and second messengers such as reactive oxygen species (ROS) and calcium (**Figure 1D**) (Aizman et al., 2001; Pierre and Xie, 2006; Cui and Xie, 2017; Trant et al., 2025). Endogenous CTS signaling at the cell membrane NKA receptor features many of the classic characteristics of G protein-coupled receptor (GPCR) signaling, including signal termination through receptor internalization upon chronic exposure to the ligand (Liu, 2005; Tian et al., 2006; Feldmann et al., 2007; Pierre et al., 2011; Gupta et al., 2012), and biased signaling (Manunta et al., 2000; Akimova et al., 2005; Liu, 2005; Zulian et al., 2013; Amaral et al., 2018; Banerjee et al., 2021; Xu et al., 2021; Blaustein and Hamlyn, 2024). Heterologous expression studies have revealed NKA α-specific characteristics of CTS signaling, which likely contribute to tissue-specific effects of eCTS (Pierre et al., 2008; Xie et al., 2015; Madan et al., 2017; Yu et al., 2018).

## Classic and novel mechanisms of sodium reabsorption regulation through the Na/K-ATPase in the renal proximal tubule

**Figure 2:**
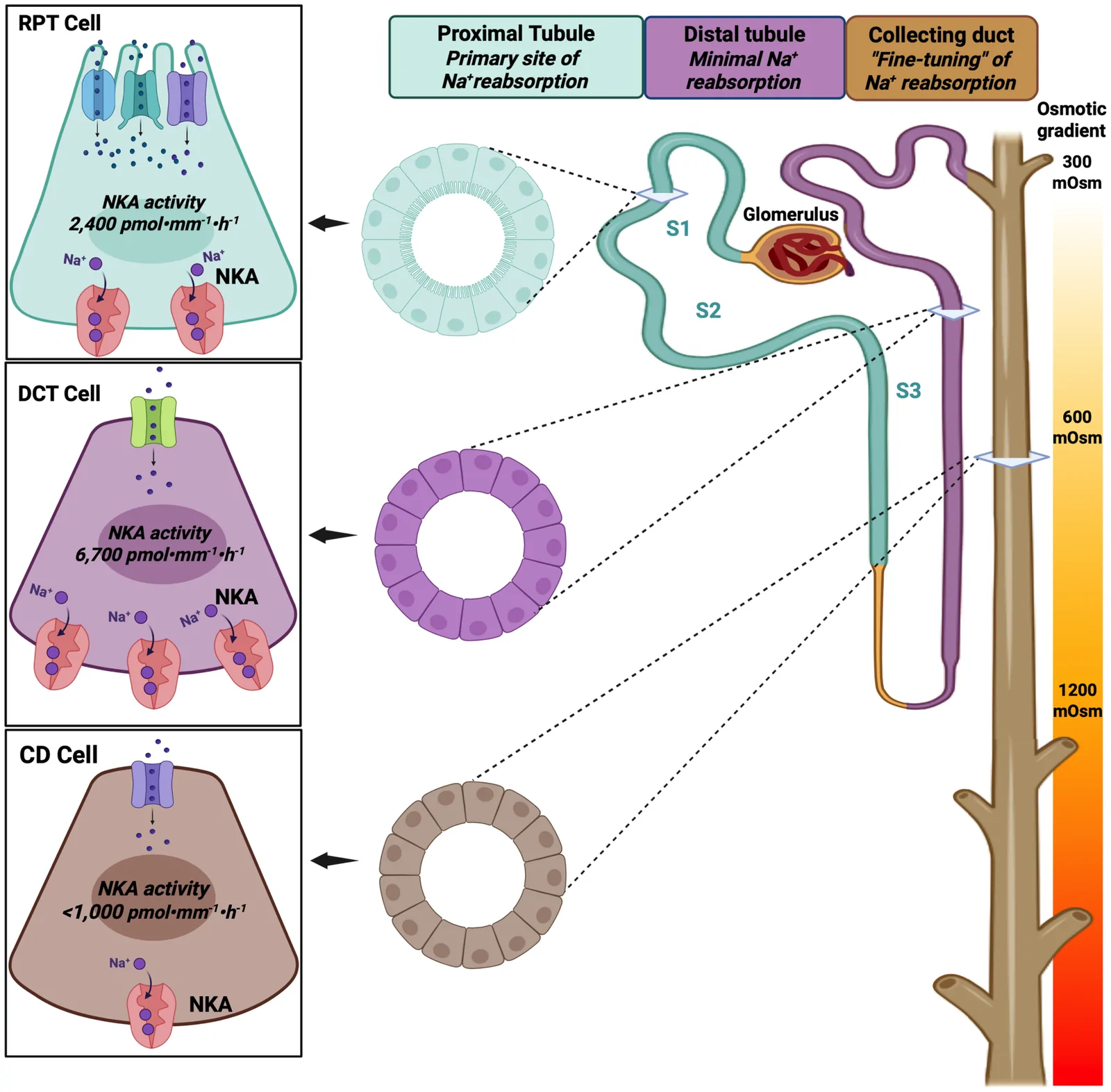
Sodium reabsorption and Na/K-ATPase (NKA) activity along the nephron. The renal proximal tubule (RPT) reabsorbs most of the filtered Na^+^ from the ultrafiltrate. Basolateral NKAs generate a Na^+^-driven electrochemical gradient to support the transport of solutes from the glomerular filtrate. In the RPT, which is divided into three segments (S1, S2, and S3) based on structural features and organellar composition, NKA maintains intermediate activity to absorb Na^+^ from filtrate which is isosmotic to the surrounding interstitia. NKA activity is highest in the distal convoluted tubule (DCT) where the filtrate becomes hypotonic, and Na^+^ absorption occurs against a concentration gradient. NKA activity is lowest in the collecting duct (CD), where “fine-tuning” of Na^+^ reabsorption takes place. Representative NKA activity values were measured as labeled phosphate released by the hydrolysis of [γ-32P] ATP in rat nephron segments (Katz et al., 1979).

The healthy adult kidney receives about 25% of the cardiac output, totaling over one liter of blood per minute. Over 90% of this supplies the renal cortex, with the remainder supplying the medulla. Human kidneys filter approximately 180 liters of plasma daily through the glomeruli’s Bowman’s capsules (Cargill, 1949; Bergström et al., 1959; Kaufman et al., 2023). The resulting ultrafiltrate then travels along the renal tubule where 99% of the solutes and water are reabsorbed (Zhuo and Li, 2013; Kaufman et al., 2023). The contribution of the proximal nephron in this process is major, with the RPT responsible for the reabsorption of about 65% of the filtered load (Hall and Guyton, 2011; Zhuo and Li, 2013; Hoenig et al., 2025). Based on cellular structural and organelle constituents, the RPT can be divided into three consecutive segmental regions, as illustrated in **Figure 2** (Maunsbach, 1966). RPT cells of S1 have a denser brush border, more mitochondria and peroxisomes, and a greater degree of basolateral interdigitation than RPT cells of the other two segments, as these features decrease along the RPT (Maunsbach, 1966; Hall et al., 2021). Functionally, more sodium-dependent reuptake occurs in S1 and S2 than S3, and this is reflected by the abundance of apical membrane transporters (Hall et al., 2021). Tubular reabsorption of filtered sodium is quantitatively the main contribution of the kidneys to salt and water homeostasis (Hoenig et al., 2025). Consequently, the modulation of vectorial sodium transport by the RPT has a major impact on sodium homeostasis, which is a key factor in the regulation of systemic blood pressure (McDonough, 2010; Zhuo and Li, 2013). Highly specialized renal sodium transport systems (NKA, channels, cotransporters, and exchangers) have been cloned and characterized in each nephron segment, providing molecular insight and druggable targets (Ali et al., 2012; Rieg and Vallon, 2018). Additionally, genetic targeting in animal models and omics continue to refine our understanding of the mechanisms regulating sodium handling in the RPT and the role of NKA in this process.

**Figure 3:**
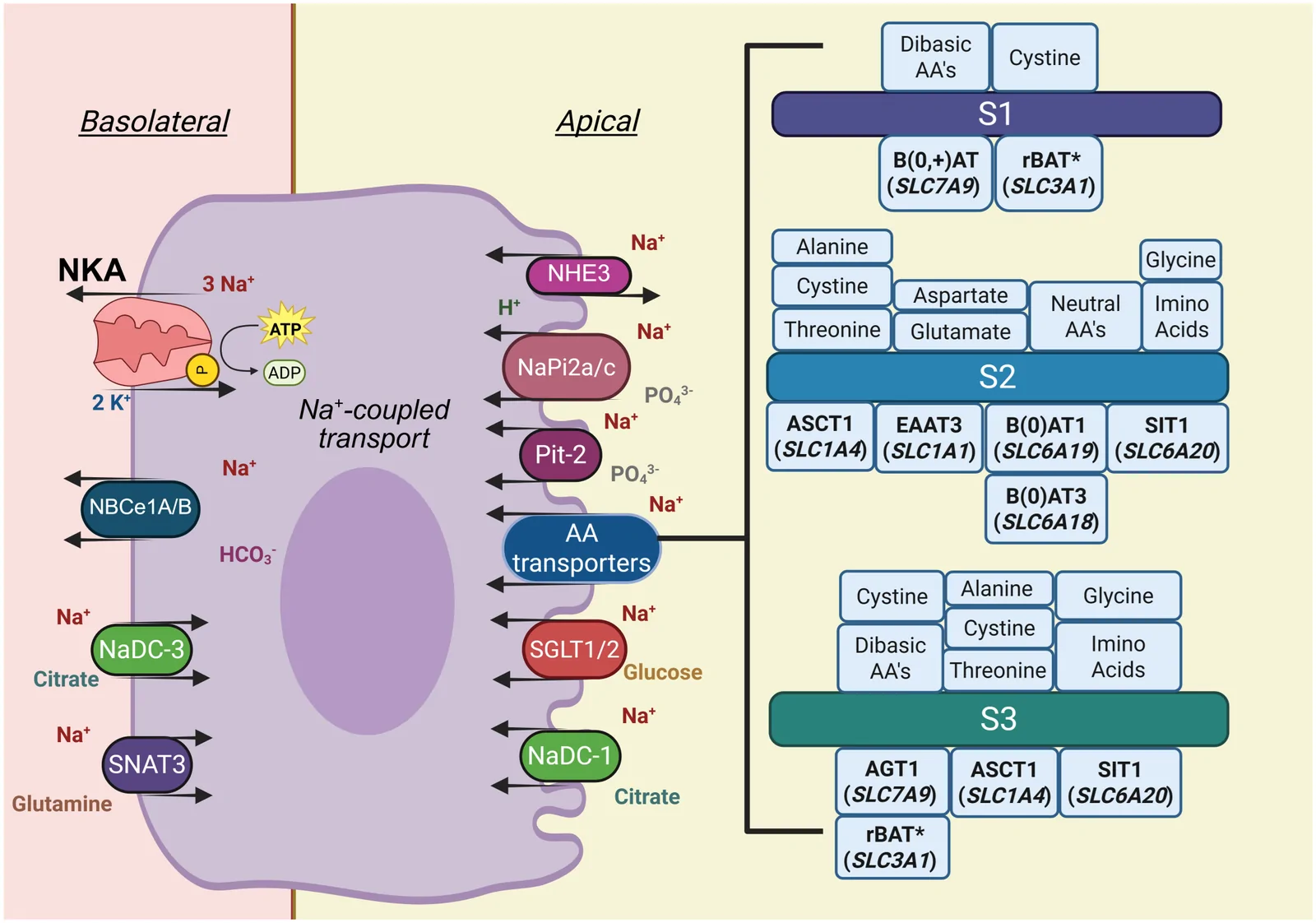
Sodium-dependent transporters of the renal proximal tubule (RPT). Na/K-ATPase (NKA) on the basolateral membrane generates a transepithelial sodium gradient that enables the net transfer of sodium from the lumen to the interstitia. Other basolateral transporters of the RPT include the sodium citrate cotransporter 3 (NaDC-3), the electrogenic sodium bicarbonate cotransporter 1A (NBCe1A), the electrogenic sodium bicarbonate cotransporter 1B (NBCe1B) in S3, and the sodium-coupled neutral amino acid transporter 3 (SNAT3). The apical sodium-dependent transporters of the RPT include the sodium hydrogen exchanger 3 (NHE3), the sodium phosphate cotransporter 2a (NaPi2a) and 2c (NaPi2c), Pit-2 (Npt3), the sodium glucose cotransporter 2 (SGLT2) in S1 and S2 segments, the sodium glucose cotransporter 1 in the S3 segment (SGLT1), and the sodium citrate cotransporter 1 (NaDC-1). In segment 1 (S1), the main Na^+^-dependent amino acid (AA) transporter is B(0,+)AT1and its auxiliary subunit rBAT. Segments 2 and 3 (S2 and S3) both contain several amino acid transporters, including ASTC1, EAAT3, B(0)AT1, B(0)AT3, SIT1, SNAT3, and AGT1 and its auxiliary subunit. rBAT. AA’s, amino acids; EAAT3, Excitatory amino acid transporter 3; ASTC1, Neutral amino acid transporter A; B(0,+)AT1-rBAT, B(0,+)-type amino acid transporter 1 and *auxiliary subunit rBAT; AGT1-rBAT, Aspartate/glutamate transporter 1 and *auxiliary subunit rBAT; B(0)AT3, Sodium-dependent neutral amino acid transporter 3; B(0)AT1, Sodium-dependent neutral amino acid transporter 1; SIT1, Sodium-dependent imino transporter 1 (aka Sodium- and chloride-dependent transporter XTRP3); SNAT3, Sodium-coupled neutral amino acid transporter 3.

NKA has long been known as a key orchestrator of renal epithelial transport. It is present in high concentration in the kidney, where its enzymatic activity far exceeds that in other organs such as the liver or the muscles (Katz, 1982; Gick et al., 1993). As illustrated in **Figure 2**, the highest NKA activity is observed in the segments which reabsorb large amounts of sodium against a concentration gradient (diluting segment and distal convoluted tubule). NKA activity is intermediate where sodium is reabsorbed from an isosmotic fluid (RPT), and low in the terminal part of the nephron where sodium reabsorption is fine-tuned (Katz et al., 1979; Féraille and Doucet, 2001). **Figure 3** illustrates the two-step process of transcellular sodium reabsorption. As in all sodium-absorbing epithelia, RPT NKA is exclusively located in the basolateral membrane, where it generates the peritubular sodium gradient that provides the driving force for passive apical entry through various sodium transport systems (Jorgensen, 1986; Féraille and Doucet, 2001). Specifically, the transepithelial sodium gradient generated by NKA, which operates only at 20–30% of its maximum rate under basal conditions, is used by the RPT membrane transporters of the apical domain (facing the nephron’s lumen) (Cheval and Doucet, 1990). This architectural organization results in a net transfer of Na^+^ from the nephron lumen toward the interstitial space. **Figure 3** summarizes the apical and basolateral Na^+^-dependent transporters described in RPT cells. RPT apical sodium transporters include the sodium hydrogen exchanger type 3 (NHE3, *SLC9A3*), the sodium phosphate cotransporter 2a (NaPi2a, also known as Napt2a*, SLC34A1*) and 2c (NaPi2c also known as Napt2c, *SLC34A3*), Pit-2 (also known as Ram-1, *SLC20A2*), the sodium glucose cotransporter 2 in the S1 and S2 segments (SGLT2, *SLC5A2*), the sodium glucose cotransporter type 1 in S3 (SGLT1, *SLC5A1*), the sodium citrate cotransporter 1 (NaDC-1, *SLC13A2*), the electrogenic sodium bicarbonate cotransporter type 2 (NBCe2, *SLC4A5*) and a number of sodium-dependent amino acid cotransporters which are detailed in **Figure 3** (Zelikovic and Chesney, 1989; Xu et al., 2003; Curthoys and Moe, 2014; Gildea et al., 2015; Palmer and Schnermann, 2015; Nagamori et al., 2016; Meier et al., 2018; Limbutara et al., 2020; Gagnon and Delpire, 2021; Lewis et al., 2021; Grubb et al., 2025). On the basolateral side, NKA is present as an α1β1 complex with the regulatory γ subunit (ATP1A1, ATP1B1, and predominately FXYD2). Other notable RPT sodium transporters expressed on the basolateral membrane include the sodium citrate cotransporter 3, also known as the sodium-dependent dicarboxylate transporter 2 (NaDC-3 or SDCT2, *SLC13A3*), the electrogenic sodium bicarbonate cotransporter type 1A (NBCe1A, *SLC4A4*), and the sodium-coupled neutral amino acid transporter 3 (SNAT3, *SLC38A3*) (Romero et al., 1997; Moret et al., 2007; Limbutara et al., 2020). The basolateral NBCe1A, which is the main pathway for bicarbonate reabsorption, is directly coupled to sodium (Skelton et al., 2010; Kurtz and Zhu, 2013). In addition, a more recently discovered splice variant, NBCe1B, is present on the basolateral membrane of the RPT, particularly in S3, where its expression is increased in response to metabolic acidosis (Fang et al., 2018).

**Figure 4:**
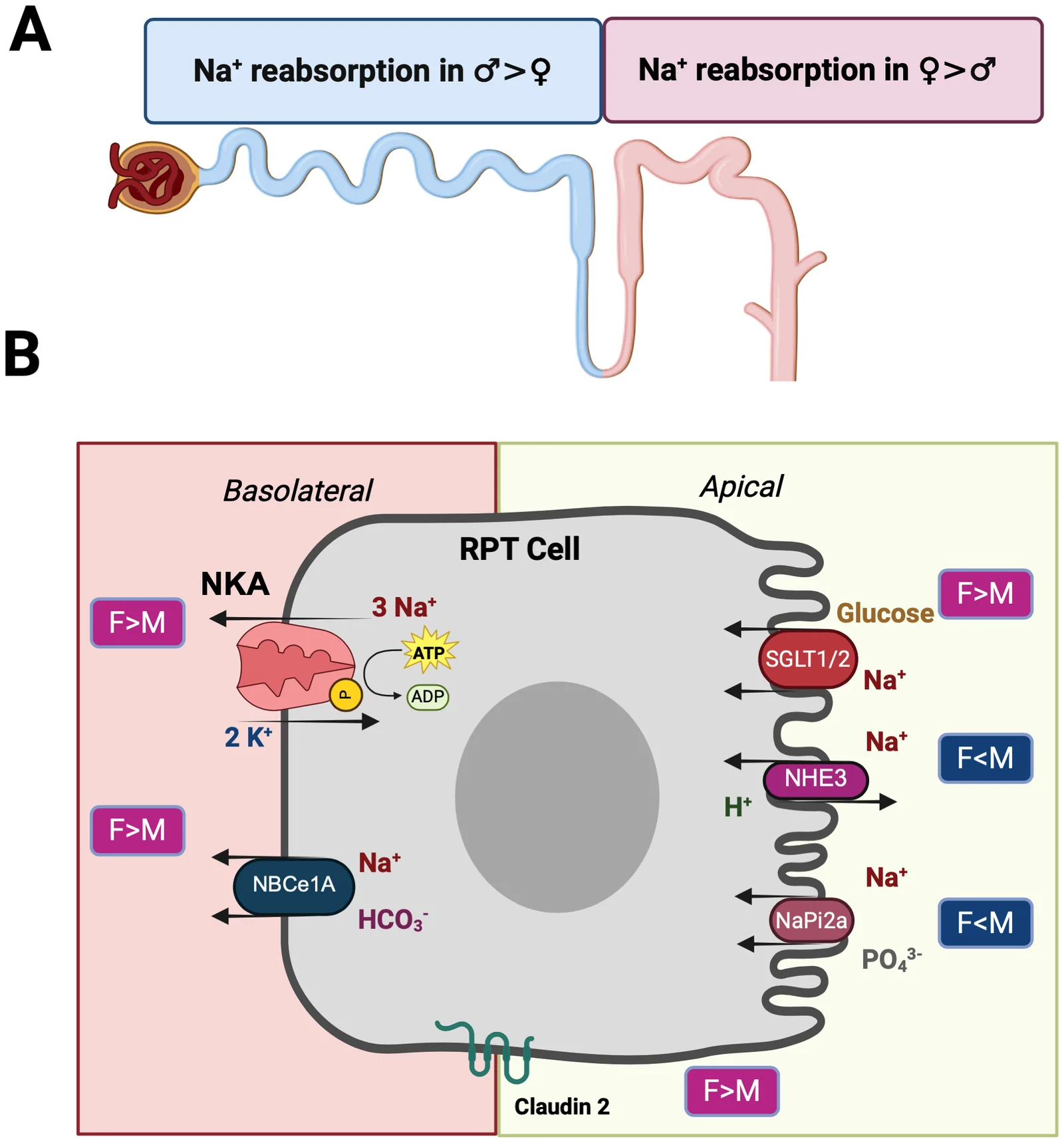
Sex differences in renal tubular transport mechanisms that regulate Na^+^ reabsorption and fluid flow along the nephron. **(A)** In males, increased expression and activity of RPT Na^+^ transporters contributes to greater Na^+^ and H_2_O reabsorption, leading to reduced Na^+^ and fluid delivery to the distal nephron compared to females. **(B)** Sexual dimorphisms of Na^+^-dependent transporters in the RPT described to date include increased expression of NHE3 and NaPi2a in males, and in females, increased expression of SGLT2 in S1 and S2 segments, SGLT1 in S3, NBCe1A, and the NKA itself, as well as Claudin 2. (Adapted from McDonough et al., 2024 and McDonough et al., 2025). NBCe1A, electrogenic sodium bicarbonate cotransporter 1A; NHE3, sodium hydrogen exchanger 3; NaPi2a, sodium-phosphate cotransporter 2a; SGLT1/2, sodium glucose cotransporter 1/2; NKA, Na/K-ATPase.

Owing to the uptick in studies including both male and female subjects, research has unveiled sex-specific differences in anatomical and molecular factors along the mammalian nephron. Sex differences include variations in tubule length, transporter abundance, and transporter activity in the RPT and other segments (Oudar et al., 1991; Veiras et al., 2017; Li et al., 2018; Huang et al., 2020; Tahaei et al., 2020; Chen et al., 2023; McDonough and Layton, 2023). Studies in rodents and computational modeling in humans suggest that females reabsorb a smaller fraction of the glomerular filtrate in the RPT, resulting in higher sodium delivery to the distal nephron (Layton and Layton, 2019; Hu et al., 2021; McDonough and Layton, 2023; McDonough et al., 2024) (**Figure 4A**). This dimorphic transporter distribution contributes to a more rapid natriuretic response to a high-salt diet in females. Consistent with a role of sex hormones, studies have shown that the androgen receptor mRNA and protein are expressed exclusively along the RPT (Harris et al., 2020), while transcripts of estrogen receptor α and β are detected in both proximal and distal tubules (Irsik et al., 2013). Additionally, evidence for gonadal hormone regulation of key renal sodium transporters and NKA have been reported (Harris et al., 2020). For example, testosterone treatment increased NHE3 activity in brush border membrane vesicles from castrated male mice, consistent with a more recent report of increased NHE3 expression in the RPT of orchiectomized males (Mačković et al., 1986; Harris et al., 2020). Importantly, using a Four Core Genotype approach that produces mouse Male XX and Female XY as well as conventional Male XY and Female XX strains, McDonough et al. recently revealed that, in addition to sex hormones, sex chromosome differences influence renal transporters expression (McDonough et al., 2025). Overall, these studies point to increased abundance of NKA α1 and β1 subunits in kidney homogenates of gonadal females compared to males (McDonough et al., 2025). Additionally, sex chromosome complementation differentially impacted abundance of NKA α1 and β1 subunits in the mouse medulla (McDonough et al., 2025). Diurnal fluxes in transporter expression in the RPT and distal tubule also differ between males and females, contributing to differential oscillations in Na^+^ excretion and blood pressure regulation (Layton and Gumz, 2022). Hence, male mice with a kidney-specific knockout of the clock gene basic helix-loop-helix ARNT like 1 (BMAL1) have lower baseline blood pressure and higher Na^+^ excretion when challenged with a K^+^-restricted diet, yet neither effect was found in female mice (Crislip et al., 2020). Males also undergo greater oscillations in Na^+^ excretion throughout the day due to a sexual dimorphism in transporters such as NHE3, based on computational modeling predictions from these studies (Layton and Gumz, 2022). Finally, sexual dimorphism in NKA and other transporters of the RPT likely contributes to sex differences observed clinically in renal disease. For example, compared to men, women are less severely affected by acute kidney injury (AKI), which is particularly impactful in the RPT due to its high metabolic rate (Grams et al., 2015; Neugarten and Golestaneh, 2022); Zhou et al., 2021; Abu Kar and Harris, 2025). Consistent with these observations, experimental work by Fekete et al. demonstrated reduced post-ischemic renal injury in female rats, with reduced membrane misdistribution of NKA α1 into the apical membrane, and greater pre- and post-ischemic expression of renal NKA α1 protein and enzymatic activity (Fekete et al., 2004). Further, in the Dahl salt sensitive rat model, a functional *Atp1a1* variant was associated with salt sensitive hypertension in males (Glorioso et al., 2007). Similarly, in a case–control genetic association study, the *ATP1A1* gene variant was linked to essential hypertension in humans, with a stronger effect in men, supporting a sex-specific effect of *ATP1A1* in blood pressure (BP) regulation (Glorioso et al., 2007). **Figure 4B** proposes a summary of the sexual dimorphism of sodium transporters described to date in the rat RPT (McDonough et al., 2025). While our understanding of sex differences in renal transporters has grown at a fast pace in the past decade, knowledge gaps remain. Building on these pioneering studies, detailed molecular studies focused on abundance, post-translational modifications, assembly of NKA αβγ complexes at the membrane, ion transport and signaling activity levels are needed to fully understand how RPT NKA may contribute to sex-specific differences in renal sodium handling.

Detailed mechanisms of the regulation of RPT sodium reabsorption through the precise control of NKA and other key transporters function by endocrine, autocrine, and neuronal factors have been reported. These regulatory mechanisms range from acute kinetic response to changes in intracellular Na^+^ concentration (Na_i_) to orchestrated transcriptional and post-transcriptional control. Besides Na_i_, the main regulatory mechanisms of RPT NKA include changes in the rate of NKA ion transport (Efendiev et al., 2002), translational and post-translational modulation of total cellular NKA abundance (namely, synthesis and degradation of NKA polypeptides) (Zhang et al., 1996; Herman et al., 2010; Taub, 2018) and cellular redistribution from/to the plasma membrane (Therien and Blostein, 2000). As reviewed in the following sections (and summarized in **Table 1**), anti-natriuretic and natriuretic hormones modulate NKA and other basolateral and apical Na^+^ transporters of the RPT through concerted but independent intracellular mechanisms downstream from their receptors, with the notable exception of eCTS.

**Table 1:** Regulators of major transporters in RPT Na^+^ handling.

Regulator	Primary receptor or target	Major signaling pathways	Main transporters affected	Principal cellular effects	Overall physiologic effect
Angiotensin II (Ang Il)	AT1-R	PLC, IP3, DAG, PKC; PI3K/Akt; Src/Raf/ERK/MAPK	NHE3, NaPi2a, NKA, NBCe1A	NHE3/NaPi2a redistribution with acute exposure NHE3 transcription with prolonged exposure NKA recruitment/activity NBCe1A activity and membrane abundance	Proximal tubular Na^+^ reabsorption
Aldosterone	MR	SGK1, EGFR	NKA, NHE3	NKA expression/activity NHE3 expression/activity	Proximal tubular Na^+^ reabsorption
Norepinephrine (NE)	⍺1/⍺2-adrenergic receptors	Ca2^+^/calcineurin; MAPK	NKA, NHE3, NBCe1A	NKA affinity for Na^+^ NHE3 activity and membrane abundance NHE3/NBCe 1A protein abundance with prolonged exposure	Proximal tubular Na^+^ reabsorption
Insulin	Insulin receptor	IRS2/PI3K; PI3K- SGK1; PKC-ζ/λ	NKA, NHE3, NBCe1A	Biphasic regulation of NKA Acute increase in NHE3 activity Chronic increase in NHE3 activity/expression NBCe 1A activity	Anti-natriuretic effect
Atrial Natriuretic Peptide (ANP)	natriuretic peptide receptor A(NPRA)	cGMP/PKG; cGMP/NO	NKA, NHE3	Inhibits NKA partly through DA signaling Augments natriuretic actions of DA Antagonizes ANG II-mediated Na^+^ reabsorption	Tubular Na^+^ reabsorption
Endothelin-1 (ET-1)	ET_B_ receptor	Ca2+/PKC; CaMK; tyrosine kinase	NKA, NHE3, NBCe1A	Inhibits NKA Stimulates NHE3/NBCe1A	Mixed natriuretic and anti-natriuretic effects
Nitric Oxide (NO)	NO/cGMP pathway	cGMP, ERK, PLC	NKA, NHE3, NBCe1A	Inhibits NKA and NBCe1A Dose-dependent regulation of NHE Overall limits sodium reabsorption	Predominantly natriuretic effect
Dopamine (DA)	D1-like receptors	PKA, PKC	NKA, NHE3, NaPi2a	NKA endocytosis NHE3 downregulation NaPi2a internalization	Na^+^ and phosphate reabsorption
Parathyroid Hormone (PTH)	PTH1R	PLC/PKC, PKA	NHE3, NaPi2a, NKA, NBCe1A	NHE3 inhibition NaPi2a internalization NKA phosphorylation/internalization Reduced bicarbonate transport	Na^+^ phosphate, and bicarbonate reabsorption
Endogenous Cardiotonic Steroids (eCTS)	NKA signaling complex	c-Src, PI3K	NKA, NHE3, NBCe1A	Coupled NKA/NHE3 signaling NKA endocytosis NHE3 downregulation Altered NBCe1A expression	Proximal tubular Na^+^ reabsorption

Renin-angiotensin-aldosterone system (RAAS). Angiotensin II (Ang II) is a main regulator of Na^+^ handling in the RPT through direct stimulatory effects on reabsorption that are independent of its effects on aldosterone and systemic hemodynamics (McDonough, 2010). AT1 receptors (AT1-R) are found on both the apical and the basolateral membranes of RPT cells. AT1-R activation triggers multiple downstream pathways, including PLC, IP_3_, and DAG, leading to increased intracellular Ca^2+^ and PKC activation (Kobori et al., 2007; Li et al., 2021; Lin et al., 2022; Tang et al., 2025). In response to acute Ang II exposure, NHE3 and NaPi2a redistribute into the apical domain of the microvilli where they are available for sodium reabsorption (Li et al., 2009; Riquier-Brison et al., 2010; Li et al., 2012), while prolonged Ang II exposure increases the transcription of NHE3 (Xu et al., 2006). Although it was initially thought that the acute stimulatory effect of Ang II on NKA was only secondary to the activation of apical sodium entry and resulting increase of Na_i_, Efendiev et al. showed that acute Ang II exposure of rat NKA α1-expressing RPT opossum kidney (OK) cells induces a PKC-dependent recruitment of NKA units to the plasmalemma (Efendiev et al., 2003). More recently, Hanna et al. showed that Ang II increases NKA activity in both rat and human RPTs through PI3K-dependent activation of Akt and phosphorylation of Ser-938 on NKA α1 (Hanna et al., 2022). Ang II further enhances RPT Na^+^ reabsorption through NBCe1A. Mechanistically, in OK cells, 0.1 nM Ang II binding rapidly increases NBCe1A activity and membrane protein abundance by activating Src kinase and the Raf/ERK/MAPK signaling pathway (Espiritu et al., 2002; Robey et al., 2002). The anti-natriuretic effects of aldosterone are mediated primarily through the renal mineralocorticoid receptors (MR) of the distal tubule and the cortical collecting duct, the hormone’s primary sites of action (Funder, 2017; Johnston et al., 2023) However, anti-natriuretic effects of aldosterone have also been reported through direct regulation of NKA and apical sodium transporters of the RPT, mediated by MR-dependent intracellular mechanisms (Drumm et al., 2006; Salyer et al., 2013).

Norepinephrine (NE) is released from sympathetic nerve endings and binds to α-adrenergic receptors in the basolateral membrane of RPT cells. Activation of α_1_-adrenergic receptors increases intracellular Ca^2+^, leading to the activation of calcineurin, a Ca^2+^/Calmodulin-dependent protein phosphatase. The activation of calcineurin increases NKA activity by increasing its affinity for Na^+^, thereby promoting Na^+^ reabsorption (Beach et al., 1987; Aperia et al., 1992; Ohtomo et al., 1996). Prolonged exposure to NE increases Na^+^ uptake by increasing the cortical protein abundance of NHE3 and NBCe1A (Sonalker et al., 2008). Both α_1_- and α_2_-adrenergic receptors increase NHE3 activity and membrane protein abundance by activating the MAPK signaling pathway (Gesek et al., 1989; Liu and Gesek, 2001; Healy et al., 2014).

Insulin. De Fronzo et al. first showed that insulin exerts an anti-natriuretic effect independently of glycemic status in healthy human (Defronzo et al., 1975). Subsequent studies revealed the molecular mechanisms of insulin-dependent regulation of RPT sodium transporters in this process. Insulin has a biphasic effect on NKA and NHE3, with stimulatory and inhibitory actions differing between the two transporters. Acute regulation of NKA occurs through both NHE3-dependent and independent mechanisms. The former involves an increase in Na_i_, while the latter enhances NKA affinity for Na^+^ via phosphorylation of Tyr-10 on the NKA α1 polypeptide (Feraille et al., 1994; Féraille et al., 1999). In OK cells, acute insulin exposure also increases apical NHE3 activity (Klisic et al., 2002). Chronic insulin exposure, however, decreases NKA activity and membrane abundance by activating PKC-ζ/λ, leading to the phosphorylation of Ser-16 and Ser-23 on NKAα1 (Banday, 2016). Chronic insulin exposure also activates NHE3 by increasing its apical activity as well as mRNA, and total and membrane protein expression through PI3K-SGK1 signaling (Klisic et al., 2002; Fuster et al., 2007). In both human and rat primary RPT cells, insulin increases NBCe1A activity, measured as the rate of pH change in response to reduced HCO_3_^-^ concentration, through insulin receptor substrate (IRS) 2/PI3K-dependent signaling (Nakamura et al., 2020).

Atrial natriuretic peptide (ANP). While the inner medullary collecting duct is the primary renal tubule target for ANP-mediated regulation of sodium handling (Zeidel et al., 1988; Theilig and Wu, 2015; Cheval et al., 2019), some of the natriuretic effects of ANP have been attributed to the inhibition of Na^+^ reabsorption by Na^+^ transporters of the RPT (Theilig and Wu, 2015). RPT NKA is inhibited by ANP through a mechanism coupled, at least in part, to dopamine (DA) signaling (Aperia et al., 1994). ANP inhibits NKA through cGMP-PKG dependent signaling that causes DA D1-like receptors to translocate from cytoplasm to the plasma membrane. The resulting increase in DA uptake decreases NKA activity, possibly through mechanisms discussed in a later section on dopamine (Brismar et al., 2000; Correa et al., 2007). ANP decreases Na^+^ reabsorption through NHE3 by 1) augmenting the natriuretic action of other hormones such as DA, or 2) by inhibiting the anti-natriuretic action of Ang II (Winaver et al., 1990; Li et al., 2024). Much of ANP’s natriuretic effects depend on the presence of Ang II. At the cellular level, ANP acts as a functional antagonist of Ang II by binding to the natriuretic peptide receptor A (NPR_A_) and activating cGMP/nitric oxide (NO) signaling to decrease tubular Na^+^ reabsorption in the RPT (Li et al., 2024).

Endothelin (ET-1) can exert natriuretic and anti-natriuretic effects on Na^+^ transport in the RPT. ET-1 reduces Na^+^ and water reabsorption primarily by inhibiting NKA through the activation of ET_B_ receptors (Liu et al., 2009). Mechanistically, NKA inhibition is Ca^2+^ and PKC-dependent (Liu et al., 2009). In contrast, ET-1 stimulation of the ET_B_ receptor activates NHE3 and NBCe1A. ET-1 stimulates NHE3 and NBCe1A in rabbit cortical membrane vesicles at a dose of 10^–10M^ by increasing their V_max_ without altering their K_m_ (Eiam-Ong et al., 1992). In OK cells, ET_B_ receptor activation increases NHE3 activity by 1) causing a rise in intracellular Ca^2+^ and activating Ca^2+^-calmodulin kinase (CaMK) and 2) through tyrosine kinase-dependent signaling (Chu et al., 1996a, 1996).

Nitric oxide (NO) is a locally produced autacoid in the RPT that limits sodium reabsorption. The natriuretic action of NO in the RPT is mediated predominantly through inhibition of NKA, NHE3, and NBCe1A (Guzman et al., 1995; Roczniak and Burns, 1996). In OK cells, NO decreases NKA activity partially by the formation of peroxynitrite radicals, as well as by activating upstream molecules such as guanylate cyclase and PLC. However, the inhibitory effect of NO on NKA may be species- or cell-type specific, as no inhibition was observed in LLC-PK1 upon PLC/PKC activation (Guzman et al., 1995; Liang and Knox, 1999). NO similarly inhibits NBCe1A by stimulating cGMP and ERK-dependent signaling (Shirai et al., 2014). While its overall effects are natriuretic, NO, and its stimulation of cGMP, has dose-dependent effects on NHE3 transport activity. Lower doses (10^–6M^) of NO increased NHE3-dependent fluid and bicarbonate reabsorption in the rat RPT, but higher doses (10^–3M^) decreased NHE3 activity in the rabbit RPT (Roczniak and Burns, 1996; Wang, 1997).

**Figure 5:**
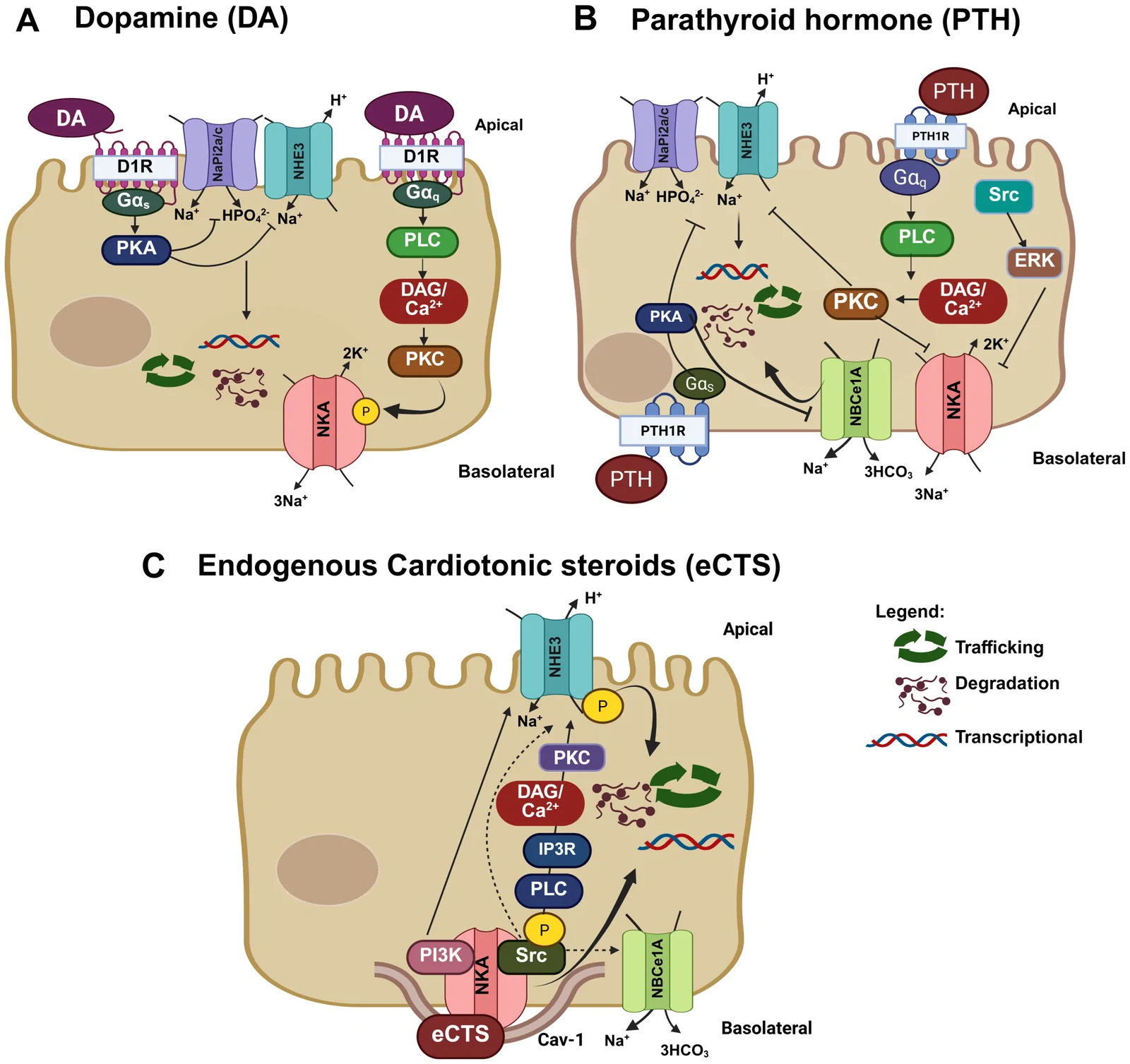
Distinct cellular mechanisms of natriuresis mediated by dopamine (DA), parathyroid hormone (PTH) and endogenous cardiotonic steroids (eCTS) **(A)** DA and **(B)** PTH act through their respective G protein–coupled receptors to activate parallel kinase pathways, with PKC-mediated inhibition of Na^+^/K^+^-ATPase (NKA) and PKA-mediated inhibition of NHE3, NBCe1A, and NaPi2a/c **(C)** eCTS binds to NKA, which functions as a signaling scaffold to initiate localized signaling cascade that are functionally coupled to inhibition of NHE3 and NKA. Cav-1, caveolin-1; DAG, diacylglycerol; PKC, protein kinase C; PLC, phospholipase C; ERK, extracellular signal-regulated kinase; PKA, protein kinase A; Src, src kinase; IP3R, inositol 1;4;5-triphosphate receptor; PI3K, phosphoinositide 3-kinase; NaPi2a/c, sodium-phosphate cotransporter 2a/c; NBCe1A, sodium bicarbonate cotransporter e1A; PTH1R, parathyroid hormone receptor 1; D1R, dopamine receptor 1; Src, Src family kinase.

Dopamine (DA). Renal DA binds to G_s_-coupled GPCR receptors (D1-like) in the RPT to reduce apical and basolateral Na^+^ transport in response to increased salt intake or volume expansion. As shown in **Figure 5A**, DA inhibits NKA through PKC-dependent phosphorylation of Ser-18, leading to its endocytosis into clathrin-coated vesicles and trafficking to early and late endosomes (Chibalin et al., 1999; Aperia, 2000). On the apical membrane, DA downregulates NHE3 through several mechanisms, including decreased translation and increased endocytosis, ubiquitination, and degradation (Hu et al., 2013). Reduced membrane abundance of NHE3 was also shown to occur through a PKA-dependent pathway that decreases both its exocytic membrane insertion and reinsertion following endocytic recycling (Hu et al., 2017). Activation of apical D1-like receptors by DA inhibits both Na^+^ and phosphate absorption by activating PKA-dependent signaling that reduces the brush border membrane expression and increases the internalization of NaPi2a (Bacic et al., 2005).

Parathyroid hormone (PTH) acts through apical and basolateral receptors (PTH1R) in the RPT. As summarized in **Figure 5B**, PTH downregulates Na^+^ reabsorption through a multimodal regulation of RPT Na^+^ transporters. PTH-mediated inhibition of NHE3 occurs by 1) acute decrease of active units following phosphorylation and redistribution away from the apical membrane and 2) long-term downregulation of protein expression and mRNA levels (Azarani et al., 1995; Girardi et al., 2000; Neri et al., 2015). PTH also causes the internalization of NaPi2a by coupling PLC and PKC-dependent signaling to NHE3 regulatory factor (NHERF)-1 (NHERF1), which interacts with the C-terminus of NaPi2a (Gisler et al., 2001; Capuano et al., 2007). PTH inhibits basolateral NKA through mechanisms that are independent of decreased apical Na^+^ transport (Zhang et al., 1999). In OK cells expressing rat NKA α1, PTH inhibits NKA by phosphorylation, inactivation, and internalization through signaling that involves Src kinase, PLC, ERK, PLA2, and PKC (Khundmiri et al., 2008). PTH also inhibits basolateral bicarbonate transport in the RPT, suggesting a negative regulation of NBCe1A. In perfused rat and rabbit RPTs, PTH administration decreased bicarbonate reabsorption and basolateral bicarbonate transport. In the rat RPT, PTH was found to inhibit basolateral bicarbonate transport through phosphatidylinositol and PKC-dependent signaling. In the rabbit RPT, inhibitory signaling was mediated by a PKA-dependent pathway (Pastoriza-Munoz et al., 1992; Ruiz et al., 1996).

Endogenous CTS (eCTS) such as ouabain or marinobufagenin have hormonal effects on natriuresis and blood pressure in mammals (Hamlyn et al., 1982; Fedorova et al., 2002; Lingrel, 2010; Fedorova et al., 2012; Shapiro et al., 2020; Blaustein and Hamlyn, 2024). Like the physiological regulators discussed above, eCTS exert their natriuretic effects, at least in part, through the modulation of the main sodium transporters in the RPT. It is important to bear in mind that the natriuretic action of eCTS occurs at concentrations that do not induce a measurable inhibition of cellular NKA activity or a significant increase of Na_i_. Hence, it is mechanistically distinct from the natriuretic effects observed upon exposure to high (inhibitory) concentrations of CTS, which are well known to induce a significant (and physiologically irrelevant) inhibition of basolateral extrusion of Na^+^ by NKA (Hook, 1969; Nechay, 1974; Eliades et al., 1991). As summarized in **Figure 5C**, the cellular signaling of eCTS in the RPT is unique among known natriuretic hormones, as it is initiated following binding to one of its RPT targets and couples to other transporters. This contrasts with DA or PTH, which act on their respective receptors to trigger a coordinated but parallel inhibition of Na^+^ transporters in the RPT. Studies in porcine RPT LLC-PK1 cells grown on Transwell inserts have revealed key features of the eCTS signaling cascade. Liu et al. first reported that the CTS ouabain, applied to the basolateral side, dose-dependently stimulates clathrin-dependent endocytosis of membrane NKA units at concentrations that do not significantly alter Na_i_ or tight junctions. Pharmacological and genetic approaches further revealed the involvement of c-Src, caveolin-1, and PI3K (Liu et al., 2004, 2005). In this cell model, acute ouabain exposure (1 h) on the basolateral side also significantly reduces apical NHE3-mediated Na^+^ transport following a redistribution of NHE3 away from the cell surface in a PI3K-, caveolin-1, c-Src-, and Ca^2+^-dependent manner (Cai et al., 2008). Chronic ouabain exposure (24 h) on the basolateral side downregulates the transcription of apical NHE3 (Oweis et al., 2006). Key characteristics of this coupled CTS/NKA/NHE3 signaling, including basolateral and apical internalization, have been confirmed in other RPT cell models (Yan et al., 2012, 2016; Mukherji et al., 2023) and in *ex vivo* and *in vivo* models (Periyasamy et al., 2005; Liu et al., 2011; Godinho et al., 2017). *Atp1a1* gene silencing in LLC-PK1 revealed additional features of the cellular signaling activated upon CTS binding to NKA. Namely, we observed that the pathway couples basolateral NKA to apical NHE3 though a mechanism that favors phosphorylation of its inhibitory site Ser-552 and alters basolateral NBCe1A expression. Specifically, when NKA expression is suppressed by 90%, despite a marked elevation (nearly doubled) of Na_i_ (Liang et al., 2007), apical ^22^Na^+^ uptake was increased, phosphorylated NHE3 Ser-552 was decreased, and NBCe1A expression was increased (Mukherji et al., 2023). Suppression of this tonic eCTS/NKA molecular natriuretic mechanism was also apparent in the renal cortex of hypomorphic mice with a 70% reduction of NKA expression in the S1/S2 RPT segments, developed using a SGLT2-Cre/Flox *Atp1a1* approach. In these mice, absolute Na^+^ excretion was decreased by 65%, leading to the conclusion that NKA signaling in the RPT is not only physiologically relevant, but also appears to be functionally dominant over NKA ion-pumping in the control of Na^+^ reabsorption in the RPT under physiological conditions (Mukherji et al., 2023).

## Significance and research prospects

Traditionally known as the enzymatic machinery that drives renal sodium reabsorption, RPT NKA has now emerged as a molecular counteractor of sodium reabsorption through its eCTS receptor function. Structurally, NKA has evolved as a perfect molecular receptor to sense Na_i_ and coordinate a cellular RPT response to temper reabsorption through signaling capabilities, some of which were acquired selectively by the mammalian NKAα1 (Liu et al., 2004; Wang et al., 2004; Tian et al., 2006; Xie et al., 2015). In humans, eCTS are present in measurable amounts under normal conditions and increase during specific physiological conditions such as exercise or pregnancy (Vakkuri et al., 2000; Schoner, 2002; Bauer et al., 2005; Jacobs et al., 2012). The physiological presence of eCTS is further supported by experimental studies employing direct and indirect approaches, including passive immunization with anti-ouabain antibody, which have demonstrated a role of eCTS in sodium homeostasis, vascular tone, cardiac hypertrophy and salt sensitive hypertension (Yamada et al., 1994; Skoumal et al., 2007; Nesher et al., 2009). Increased eCTS excretion has been linked to enhanced RPT-mediated fractional Na^+^ excretion and has also been shown to be inversely related to age and age-dependent increase in salt sensitivity (Anderson et al., 2008). Plasma levels of eCTS are also elevated in several diseases, including chronic kidney disease, hyperaldosteronism, essential hypertension, congestive heart failure, and preeclampsia (Gottlieb et al., 1992; Kolmakova et al., 2011; Nikitina et al., 2011; Simonini et al., 2015; Blaustein and Hamlyn, 2024). In this context, it is important to bear in mind that the physiological and pathological responses to eCTS binding to NKA receptors are systemic and multicellular. This is well documented for CTS regulation of renal sodium handling, which is not mediated exclusively through NKAs of the RPT. Rather, there is ample evidence to support cell-specific responses of NKAs along the renal tubule, as well as extra-renal NKAs (Schoner and Scheiner-Bobis, 2008; Fedorova et al., 2010; Blaustein and Hamlyn, 2024). Notably, this integrated natriuretic response is also modulated through tissue-specific regulation of NKA by other hormones (e.g, ANP) (Fedorova et al., 2006) There is also ample experimental support for a complex and multimodal role of eCTS in essential hypertension (Feijó and Quintas, 2025). In this context, a dysfunction of the RPT NKA receptor under chronic exposure to eCTS and oxidative stress occurs, with significant contribution to the deterioration of renal sodium handling and blood pressure (Liu et al., 2011; Kennedy et al., 2013; Yan et al., 2013; Shapiro et al., 2020). Hence, there is experimental support for a therapeutic approach aiming at normalizing RPT NKA receptor function (Xiao et al., 2018; Cheng et al., 2019; Li et al., 2023).

Beyond sodium handling, the RPT NKA receptor function has been shown to modulate cell viability. For example, through RPT NKA/IP3R activation, the CTS ouabain has been shown to protect RPT cells from apoptosis induced by high glucose (Bernhem et al., 2021). RPT NKA signaling was also protective in hyperuricemia-induced tubular injury, in which uric acid-induced injury in human RPT cells was associated with decreased NKA activity and membrane NKA α1 expression. In this model, overexpression of NKAα1 decreased injury and normalized Src/NLRP3/IL-1β signaling (Xiao et al., 2018). Finally, in primary rat RPT cells, ouabain administration activates NKA/IP3 signaling to prevent apoptosis in proteinuric kidney disease (Burlaka et al., 2016). While further research is required to elucidate the subcellular pathways that link basolateral RPT NKA, NBCe1A and apical NHE3, possible coupling between RPT NKA and other sodium-coupled transporters of the RPT remain largely unexplored. These knowledge gaps have significant implications for prevalent conditions and disorders of RPT cells. Indeed, due to their high oxygen and energy demands, RPT cells are highly susceptible to injury from hyperfiltration and/or nephrotoxins (Chevalier, 2016; Xiao et al., 2018; Ho and Morgan, 2022). These include renal wasting diseases such as Fanconi Syndrome, which has been associated with decreased RPT NKA expression and activity (Castaño et al., 1997). RPT cells are also vulnerable to drug toxicity from medications such as cisplatin and zoledronate and ischemic acute kidney injury (Markowitz et al., 2003; Schaub et al., 2021). Hence, nearly seven decades after J. C. Skou’s pioneering discovery, extensive insight has been gained into the structure and function of the NKA membrane complex. Nonetheless, knowledge from gene polymorphism, biased signaling, and sex-specific characteristics of RPT NKA classic and novel functions will likely continue to advance our understanding of its role in health and disease.

## Glossary

Ang II: Angiotensin II, ANP, Atrial natriuretic peptide
AT1-R: AT1 receptors
ATP: Adenosine triphosphate
BP: blood pressure
cAMP: cyclic adenosine monophosphate
cGMP: cyclic guanosine monophosphate
CTS: cardiotonic steroids
DA: Dopamine
DAG: diacylglycerol
eCTS: endogenous cardiotonic steroids
ERK: extracellular signal-regulated kinase
ET-1: Endothelin-1
GFR: glomerular filtration rate
GPCR: G protein-coupled receptor
HFrEF: heart failure with reduced ejection fraction
IL-1: interleukin-1
IP3: inositol 1;4;5-trisphosphate
IRS: insulin receptor substrate
MAPK: mitogen-activated protein kinase
NE: Norepinephrine
NKA: Na/K-ATPase
NLRP3: NLR family pyrin domain containing 3
NO: nitric oxide
NPRA: natriuretic peptide receptor A
OK: opossum kidney
PI3K: phosphoinositide 3-kinase
PKA: protein kinase A
PKC: protein kinase C
PLA2: phospholipase A2
PLC: phospholipase C
PLM: phospholemman
PP1: protein phosphatase-1
PTH: Parathyroid hormone
ROS: reactive oxygen species
RPT: renal proximal tubule
MR: Mineralocorticoid receptor
SGK1: (Serum/Glucocorticoid-regulated Kinase 1)
EGFR: Epidermal Growth Factor Receptor
